# Correction: Computational phenotypes for patients with opioid-related disorders presenting to the emergency department

**DOI:** 10.1371/journal.pone.0324877

**Published:** 2025-05-16

**Authors:** R. Andrew Taylor, Aidan Gilson, Wade Schulz, Kevin Lopez, Patrick Young, Sameer Pandya, Andreas Coppi, David Chartash, David Fiellin, Gail D’Onofrio

In the Study design and setting subsection of Materials and methods, there is an error in the sixth sentence. The correct sentence is: The Yale institutional review board approved this study and waived the need for informed consent (HIC# 2000025279).
