# Supplementary material for: Computational phenotypes for patients with opioid-related disorders presenting to the emergency department
Source: PLoS One. 2023 Sep 15;18(9):e0291572. doi: 10.1371/journal.pone.0291572 (PMC10503758; doi:10.1371/journal.pone.0291572)
Supplement: S2 Table — (DOCX) [file pone.0291572.s002.docx]

S2 Table

| Abreviation | Code | Name |
| --- | --- | --- |
| aggp | T100 | Age Group |
| antb | T195 | Antibiotic |
| bacs | T123 | Biologically Active Substance |
| bhvr | T053 | Behavior |
| clna | T201 | Clinical Attribute |
| clnd | T200 | Clinical Drug |
| diap | T060 | Diagnostic Procedure |
| dsyn | T047 | Disease or Syndrome |
| hcro | T093 | Health Care Related Organization |
| hlca | T058 | Health Care Activity |
| lbpr | T059 | Laboratory Procedure |
| lbtr | T034 | Laboratory or Test Result |
| medd | T074 | Medical Device |
| neop | T191 | Neoplastic Process |
| ocac | T057 | Occupational Activity |
| ocdi | T090 | Occupation or Discipline |
| phsu | T121 | Pharmacologic Substance |
| socb | T054 | Social Behavior |
| sosy | T184 | Sign or Symptom |
| topp | T061 | Therapeutic or Preventive Procedure |
