# Supplementary material for: Computational phenotypes for patients with opioid-related disorders presenting to the emergency department
Source: PLoS One. 2023 Sep 15;18(9):e0291572. doi: 10.1371/journal.pone.0291572 (PMC10503758; doi:10.1371/journal.pone.0291572)
Supplement: S3 Table — The opioid cohort was older with a mean age of 48.7 vs 42.0 (p < 0.001) for the non-opioid cohort. More predominantly male, 53.8% vs 45.9% (p < 0.001), and more predominantly Caucasian 65.0% vs 53.4% (p < 0.001). (DOCX) [file pone.0291572.s003.docx]

S3 Table:

|  |  | Overall | No Opioid Event | Opioid Exposure | P-Value |
| --- | --- | --- | --- | --- | --- |
| n |  | 169207 | 86630 | 82577 |  |
| Age(years), mean (SD) |  | 45.3 (21.4) | 42.0 (25.4) | 48.7 (15.6) | <0.001 |
| Sex, n (%) | Female | 84987 (50.2) | 46884 (54.1) | 38103 (46.1) | <0.001 |
| Sex, n (%) | Male | 84196 (49.8) | 39740 (45.9) | 44456 (53.8) |  |
| Sex, n (%) | Unknown | 24 (0.0) | 6 (0.0) | 18 (0.0) |  |
| Race, n (%) | White or Caucasian | 99924 (59.1) | 46252 (53.4) | 53672 (65.0) | <0.001 |
| Race, n (%) | Black or African American | 38002 (22.5) | 20952 (24.2) | 17050 (20.6) |  |
| Race, n (%) | Other | 26850 (15.9) | 16041 (18.5) | 10809 (13.1) |  |
| Race, n (%) | Asian | 1884 (1.1) | 1627 (1.9) | 257 (0.3) |  |
| Race, n (%) | Unknown | 1598 (0.9) | 1255 (1.4) | 343 (0.4) |  |
| Race, n (%) | American Indian or Alaska Native | 581 (0.3) | 286 (0.3) | 295 (0.4) |  |
| Race, n (%) | Native Hawaiian or Other Pacific Islander | 368 (0.2) | 217 (0.3) | 151 (0.2) |  |
| Ethnicity, n (%) | Non-Hispanic | 133288 (78.8) | 65506 (75.6) | 67782 (82.1) | <0.001 |
| Ethnicity, n (%) | Hispanic or Latino | 34912 (20.6) | 20433 (23.6) | 14479 (17.5) |  |
| Ethnicity, n (%) | Unknown | 1007 (0.6) | 691 (0.8) | 316 (0.4) |  |
