# Supplementary figures and images for: Computational phenotypes for patients with opioid-related disorders presenting to the emergency department
Source: PLoS One. 2023 Sep 15;18(9):e0291572. doi: 10.1371/journal.pone.0291572 (PMC10503758; doi:10.1371/journal.pone.0291572)

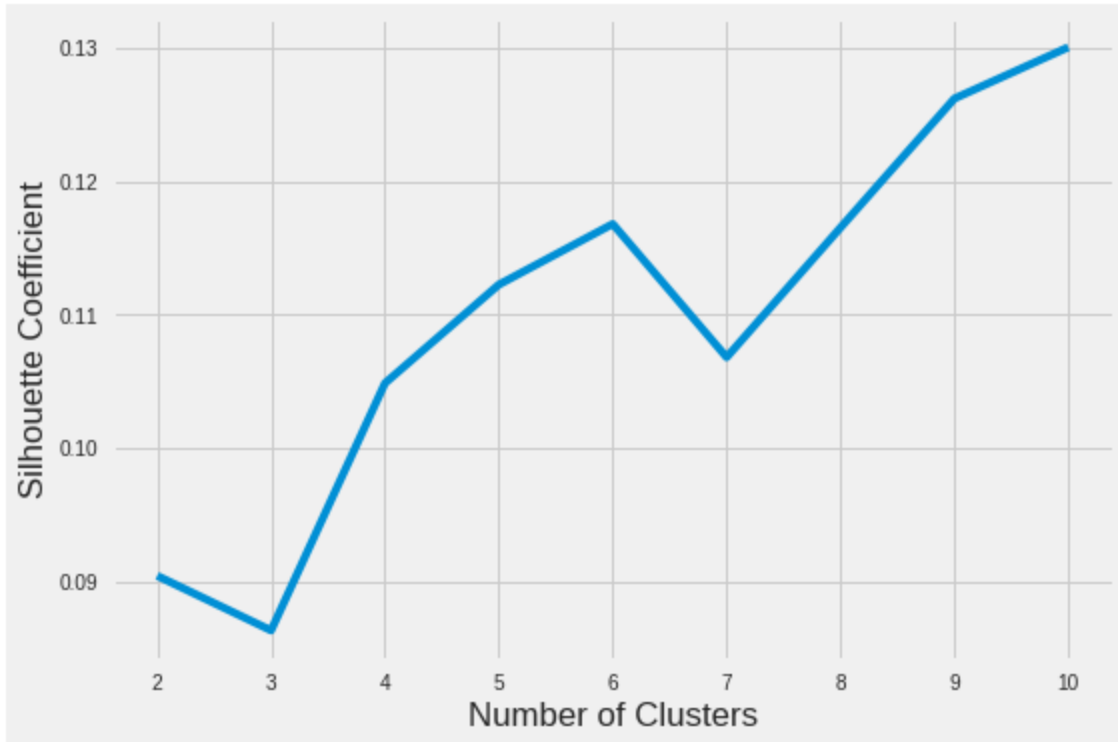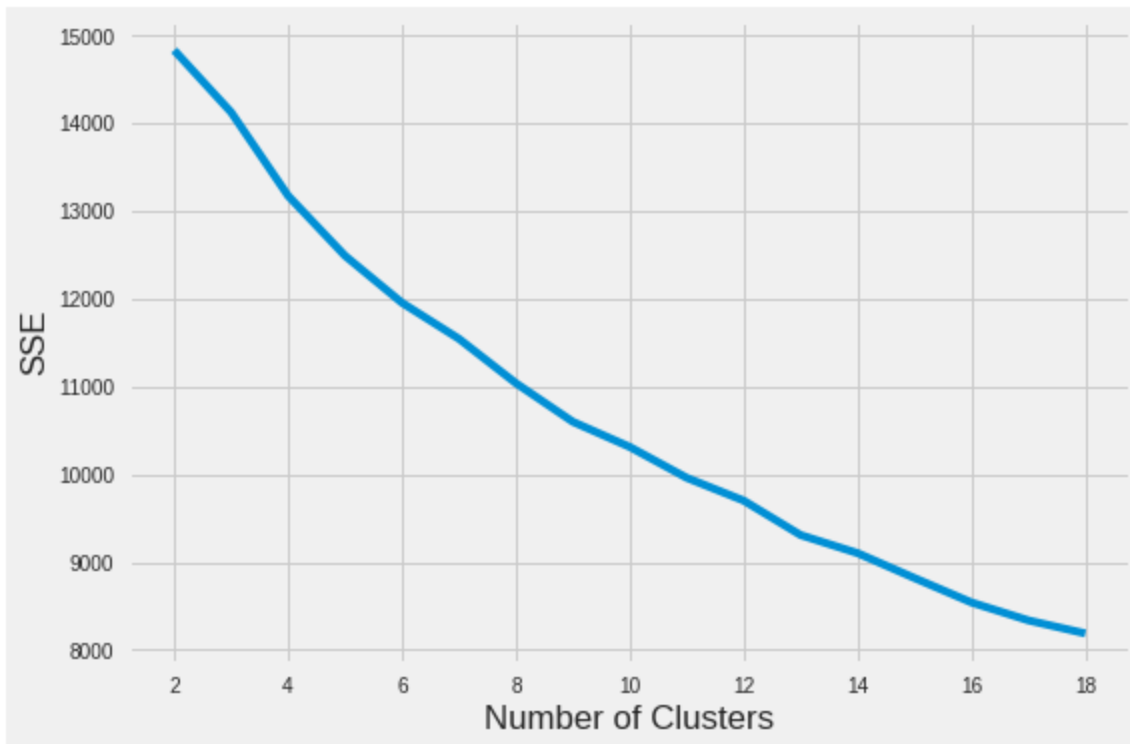

Supplement: S2 Fig — (PDF) [file pone.0291572.s005.pdf]
