# Supplementary material for: Computational phenotypes for patients with opioid-related disorders presenting to the emergency department
Source: PLoS One. 2023 Sep 15;18(9):e0291572. doi: 10.1371/journal.pone.0291572 (PMC10503758; doi:10.1371/journal.pone.0291572)

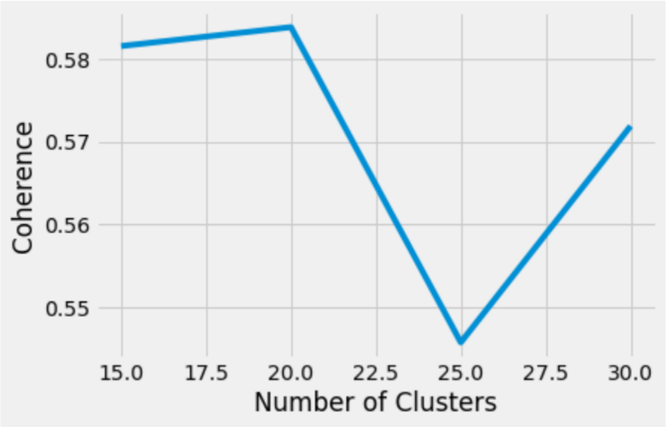
Coherence
0.58
0.57
0.56
0.55
15.0
17.5
20.0
22.5
25.0
27.5
30.0
Number of Clusters

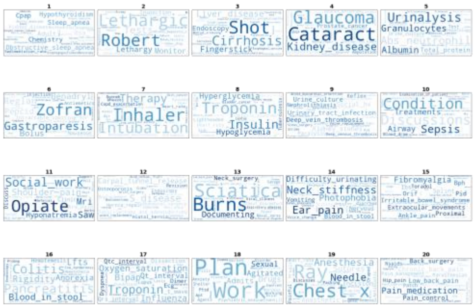

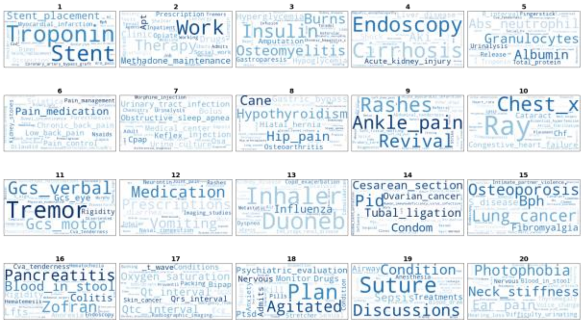

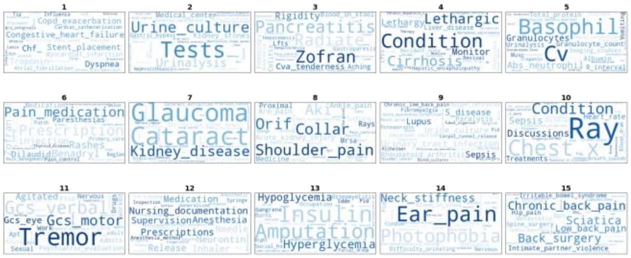

Supplement: S1 Fig — (PDF) [file pone.0291572.s004.pdf]
